# CD47xCD19 bispecific antibody triggers recruitment and activation of innate immune effector cells in a B-cell lymphoma xenograft model

**DOI:** 10.1186/s40164-022-00279-w

**Published:** 2022-05-10

**Authors:** Xavier Chauchet, Laura Cons, Laurence Chatel, Bruno Daubeuf, Gérard Didelot, Valéry Moine, Didier Chollet, Pauline Malinge, Guillemette Pontini, Krzysztof Masternak, Walter Ferlin, Vanessa Buatois, Limin Shang

**Affiliations:** 1 Light Chain Bioscience/Novimmune S.A, 15 Chemin du Pré-Fleuri, 1228 Plan-les-Ouates, Switzerland; 2 iGE3 Genomics Platform, CMU-University of Geneva, Geneva, Switzerland

**Keywords:** Cancer immunotherapy, CD47, Bispecific antibody, B-cell lymphoma, Tumor microenvironment, Macrophages, NK cells, Dendritic cells

## Abstract

**Background:**

CD47/SIRPα axis is recognized as an innate immune checkpoint and emerging clinical data validate the interest of interrupting this pathway in cancer, particularly in hematological malignancies. In preclinical models, CD47/SIRPα blocking agents have been shown to mobilize phagocytic cells and trigger adaptive immune responses to eliminate tumors. Here, we describe the mechanisms afforded by a CD47xCD19 bispecific antibody (NI-1701) at controlling tumor growth in a mouse xenograft B-cell lymphoma model.

**Methods:**

The contribution of immune effector cell subsets behind the antitumor activity of NI-1701 was investigated using flow cytometry, transcriptomic analysis, and in vivo immune-cell depletion experiments.

**Results:**

We showed that NI-1701 treatment transformed the tumor microenvironment (TME) into a more anti-tumorigenic state with increased NK cells, monocytes, dendritic cells (DC) and MHCII^hi^ tumor-associated macrophages (TAMs) and decreased granulocytic myeloid-derived suppressor cells. Notably, molecular analysis of isolated tumor-infiltrating leukocytes following NI-1701 administration revealed an upregulation of genes linked to immune activation, including IFNγ and IL-12b. Moreover, TAM-mediated phagocytosis of lymphoma tumor cells was enhanced in the TME in the presence of NI-1701, highlighting the role of macrophages in tumor control. In vivo cell depletion experiments demonstrated that both macrophages and NK cells contribute to the antitumor activity. In addition, NI-1701 enhanced dendritic cell-mediated phagocytosis of tumor cells in vitro, resulting in an increased cross-priming of tumor-specific CD8 T cells.

**Conclusions:**

The study described the mechanisms afforded by the CD47xCD19 bispecific antibody, NI-1701, at controlling tumor growth in lymphoma mouse model. NI-1701 is currently being evaluated in a Phase I clinical trial for the treatment of refractory or relapsed B-cell lymphoma (NCT04806035).

**Supplementary Information:**

The online version contains supplementary material available at 10.1186/s40164-022-00279-w.

## Background

It has emerged that tumor-infiltrating innate immune cells could also be therapeutically targeted in cancer [1]. Indeed, tumor-associated macrophages (TAMs), a generally abundant population in tumors, have been associated with cancer dissemination, immune suppression and resistance to treatment in multiple cancers [2, 3]. Consequently, treatments have been developed to limit TAMs accumulation or to reprogram them to utilize their killing properties and favor antitumor response [4–8]. One of these approaches consists of harnessing the phagocytic activity of the TAMs by targeting the CD47/SIRPα immune checkpoint [9]. CD47 is a ubiquitous membrane protein that interacts with the inhibitory signal-regulatory protein alpha (SIRPα) receptor expressed by myeloid cells, and recently discovered on activated NK cells [10]. The interaction, known as a “don’t eat me” signal, contributes to tissue homeostasis by negatively regulating phagocytosis of healthy cells by macrophages but could also prevent NK-cell mediated killing [9–11]. Remarkably, previous studies have shown that a high CD47 expression on tumor cells is associated with poor prognosis in both hematological and solid cancers [12–14]. Thus, several CD47/SIRPα axis inhibitors have been developed and recently entered into clinical trials [15]. Preliminary results unveiled encouraging clinical activity of a humanized anti-CD47 monoclonal antibody (magrolimab, formerly known as 5F9-G4), in combination with standard-of-care, in refractory/relapsed follicular lymphoma and DLBCL, acute myeloid leukemia and myelodysplastic syndrome [16, 17].

There are, however, negative consequences of non-specifically targeting CD47. First, due to the broad expression of CD47 on blood cells, including erythrocytes and platelets, clinical development of Fc-active molecules are hindered by hematological dose-limiting toxicity [18]. To circumvent this issue, most molecules in clinical trials have been engineered with an IgG4 or a mutated IgG1 Fc variant to limit or inactivate Fc-effector function, reducing their toxicity but also reducing their antitumor activity as monotherapy [19–21]. As such, these molecules are likely to be effective in combination with fully competent FcγR-engaging antibodies to tumor-associated antigens or with agents promoting the expression of prophagocytic signal on tumor cells [16, 17, 19, 20]. Second, blood cells represent a large CD47-antigen sink which reduces drug availability for tumor cells and, thus, requires the administration of high maintenance doses to achieve saturation of CD47 [22].

As an approach to selectively target CD47 on cancer cells, we have developed a fully human IgG1 bispecific antibody (bsAb) platform, which takes advantage of the fully competent Fc effector functions of human IgG1 and avoids on-target/off-tumor toxicity of CD47 [23]. In preclinical studies, NI-1701 triggered potent antibody-dependent cellular phagocytosis (ADCP) and cytotoxicity (ADCC) of tumor B cells in vitro*,* through co-engagement of CD47 and CD19, and controlled tumor progression of B-cell lymphoma and leukemia xenograft models [24]. Importantly, NI-1701 demonstrated no deleterious effect on hematologic parameters and a favorable pharmacokinetic profile following administration to non-human primates [24].

In the present study, we investigated the in vivo mechanism of action of NI-1701 by focusing on its impact on the tumor microenvironment (TME) using a Burkitt’s lymphoma xenograft model and described the beneficial contribution of immune cell subsets to the antitumor activity.

## Material and methods

### Animals, cell lines and reagents

6- to 10-week-old female CB17-SCID, NOD SCID and BALB/c mice were purchased from Charles River Laboratories (Saint-Germain-Nuelles, France). Animal experiments were approved by the animal research committee of Geneva canton and experiments performed in accordance with the Swiss Federal Veterinary Office guidelines. CL-4 transgenic BALB/c mice with Hemagglutinin(HA)-specific TCR expressed by CD8^+^ T cells [25] were kindly provided by Dr. Roland Liblau (Research Center Toulouse Purpan, CPTP-INSERM).

The Burkitt’s lymphoma Raji cell line (CCL-86) was purchased from ATCC and cultured in RPMI 1640 (Sigma-Aldrich) supplemented with 10% heat-inactivated fetal calf serum (FCS, Invitrogen) and 2 mM l-glutamine (Sigma-Aldrich). The Raji GFP^hi^ cell line was generated by transfecting Raji cells with the randomly inserted GFP transgene (UniprotKB-C5MKY7) to allow the analysis of tumor cell uptake by phagocytes. The Raji HA-GFP cell line was developed by electroporating hemagglutinin (HA) gene from influenza virus strain A/Puerto Rico/8/1934 H1N1^+^ (UniProtKB-P03452) and GFP sequence integrated in an IRES-containing bicistronic vector. The transgene was inserted by targeting specifically the human Rosa26 locus using the CRISPR-Cas9 system. Stably expressing pool was enriched by successive flow cytometry cell sorting of GFP positive cells (Beckman Coulter MoFlo Astrios) and clones were generated by single cell sorting.

The generation and characterization of NI-1701, a fully human IgG1 anti-CD47xCD19 bsAb with unbalanced affinity towards CD19 (K_D_ = 0.6 nM) and CD47 (K_D_ = 500 nM), was previously described [24]. Human IgG1 isotype-matched control mAb was produced and purified at Light Chain Bioscience/Novimmune from Chinese Hamster Ovary (CHO) cell culture supernatants.

### Raji Burkitt lymphoma xenograft model

NOD SCID mice were injected subcutaneously at the flank with 5 × 10^6^ Raji GFP^hi^ cells. Tumors were measured three times a week using a digital caliper and tumor volume determined using the formula (width × length × height) × π/6. NI-1701 or human IgG1 isotype-matched control mAb were administered by intravenous (i.v.) (lateral tail vein) or intraperitoneal (i.p.) injection on a weekly basis, at a dose of 20 mg/kg, when tumor volume reached about 100 mm^3^.

### Tumor and spleen dissociation protocol

Mice were euthanized by CO_2_ inhalation and tumors or spleens directly excised and preserved in RPMI. Tumors were cut into small pieces using surgical scissors and further enzymatically and mechanically dissociated using the Gentle MACS dissociator (Miltenyi Biotec), following the recommendations provided by the human Tumor Dissociation Kit (Miltenyi Biotec). Spleens were incubated in collagenase IV (Gibco) and DNase I (Sigma-Aldrich) and mechanically dissociated using the Gentle MACS dissociator. After a washing step, red blood cells were lysed using ACK (Ammonium–Chloride–Potassium) buffer and cellular suspensions were filtered through a 70 µm cell strainer, washed and suspended in FACS buffer (PBS, BSA 1%, EDTA 2 mM) to obtain a homogeneous single-cell suspension for flow cytometry analysis.

### Flow cytometry, imaging flow cytometry and cell sorting

The following anti-mouse Abs or viability dyes were used for staining: anti-Ly6C (HK1.4), anti-CD11c (N418) and anti-CD206 (C068C2) purchased from Biolegend; anti-CD45 (30F11), anti-Ly6G (1A8), anti-CD11b (M1/70), anti-CD8a (53-6.7), anti-CD80 (16-10A1), anti-CD86 (GL1), anti-MHC Class I H-2Kd (SF1-1.1), anti-MHC Class II I-A/I-E (M5/114.15.2) and Fixable Viability Stain 620 purchased from BD Biosciences; anti-F4/80 (BM8), anti-NKp46 (29A1.4), anti-MHC II I-Ad (AMS-32.1), anti-SIRPα (P84) and LIVE/DEAD Fixable Violet Dead Cell Stain purchased from Thermo Fischer Scientific.

Cellular suspensions were first stained with viability dye (to exclude dead cells) for 20 min at room temperature following the manufacturer’s protocol. After 2 washes in FACS buffer, cells were incubated with purified rat anti-mouse CD16/32 Ab (Mouse BD Fc Block) for 5 min at 4 °C to block non-specific labeling. Then samples were directly processed for antibody staining for 20 min at 4 °C, washed 2 times and fixed in BD CellFIX (BD Biosciences). Data were acquired using a CytoFLEX S flow cytometer (Beckman Coulter, Indianapolis, IN, USA) and analysed with FlowJo software (FlowJo, LLC).

In vivo phagocytosis was determined by flow cytometry after tumor dissociation by detection of GFP in F4/80^+^ tumor-associated macrophage or F4/80^−^CD11c^+^MHCII^+^ dendritic cell (DC) populations and validated using FlowSight imaging flow cytometer (Luminex Corp.) (see more details in Additional file 2).

For isolation of tumor-infiltrating mouse leukocytes, single-cell suspensions obtained from excised tumors were submitted to a double-step sorting. First, mouse CD45^+^ cells were enriched using CD45 (TIL) MicroBeads (Miltenyi biotec) following the manufacturer’s protocol. Then, after a 5 min staining with TOPRO-3 reagent to exclude dead cells (TOPRO-3^+^), the viable tumor-infiltrating mouse leukocytes (CD45^+^, TOPRO-3^−^) were sorted using a S3e cell sorter (Bio-Rad).

HA-specific TCR CD8^+^ T cells were isolated from spleens of CL-4 transgenic mice. Briefly, spleens were dissociated and digested as described above and the single cell suspension was processed using the EasySep™ Mouse CD8^+^ T Cell Isolation Kit (STEMCELL Technologies) following the manufacturer’s instructions.

### Nanostring nCounter targeted transcriptomic analysis of tumor-infiltrating mouse leukocytes

Total RNA was isolated from purified tumor-infiltrating mouse leukocytes using ReliaPrep™ RNA Cell Miniprep System (Promega) followed by DNAse treatment with DNA-free DNA Removal kit (Thermo Fischer Scientific). Purified total RNA was quantified by Qubit (Thermo Fischer Scientific) and checked for quality by the Bioanalyzer RNA 6000 Nano assay (Agilent Technologies). Gene expression was quantified with the NanoString nCounter platform using 100 ng of total RNA in the nCounter® Mouse Myeloid Innate Immunity panel v2 (NanoString Technologies). Briefly, the code set was hybridized with the purified RNA overnight at 65 °C. RNA transcripts were immobilized and counted using the NanoString nCounter Digital Analyzer. Normalized raw expression data were analysed when two SDs above the geometric mean of the code-set-internal negative control probes were reached. The 557 remaining genes after background filtering were normalized to the geometric mean of 18 housekeeping genes included in the panel and were log2-transformed for further analysis.

### Macrophage and NK cell depletion in Raji Burkitt lymphoma xenograft model

For macrophage depletion experiment, CB17-SCID mice were injected s.c. into the flank with 5 × 10^6^ Raji cells and concomitantly treated with a loading dose (i.v., lateral tail vein) of 100 μL of clodronate or control (PBS) liposomes (Liposoma B.V., The Netherlands), followed by maintenance doses of 50 μL, every 3 or 4 other days, until the end of the experiment.

For NK cell depletion, NOD SCID mice were injected s.c. into the flank with 5 × 10^6^ Raji GFP^hi^ cells and one week later treatments were initiated with 50 µg of anti-asialo GM1 Ab (Thermo Fischer Scientific) or PBS, with a weekly injection, until the end of the experiment.

### Antibody dependent cellular phagocytosis assay with bone-marrow derived dendritic cells (BMDCs)

Single-cell suspensions of bone marrow cells were obtained from femur and tibia of BALB/c mice. Red blood cells were lysed using ACK buffer and then cell suspension was filtered with a 70 µm cell strainer. 10 million of bone-marrow cells were plated in 10-cm Petri dishes (day 0) and cultured in RPMI 1640 medium containing 10% heat-inactivated FCS, 2 mM l-glutamine, 1 mM sodium pyruvate, 10 mM HEPES buffer, 50 μM 2-mercaptoethanol and 25 μg/mL gentamicin (Sigma-Aldrich) supplemented with 20 ng/mL of GM-CSF (Peprotech). At day 4 or 5, non-adherent cells were collected and pooled with adherent cell fraction detached with Trypsin–EDTA. Cells were resuspended in fresh medium with 20 ng/mL of GM-CSF and 10 million of cells were plated per Petri dishes. At day 10, BMDCs (non-adherent cell fraction) were harvested (whereas adherent cells were discarded) and characterized by flow cytometry.

Phagocytosis experiments were performed in ultra-low attachment plates (Corning/Sigma-Aldrich) by mixing Raji GFP^hi^ cells with BMDCs (effector to target cells ratio of 1:1) in presence of 10 μg/mL of NI-1701 or hIgG1 control Ab. After 2.5 or 24 h of incubation at 37 °C, cells were harvested, suspended and stained with viability marker and anti-CD11c antibody and analysed by flow cytometry. CD11c^+^GFP^+^ double-positive events were identified as phagocytosis events, as confirmed by imaging flow cytometry (FlowSight, Luminex Corp.).

### Antigen cross-presentation assay

First, an overnight phagocytosis step was performed by co-culturing 5 × 10^4^ BMDCs with Raji HA-GFP cells (E:T ratio 1:1) in presence of 10 μg/mL of NI-1701 or hIgG1 control Ab. Then, purified HA-specific CD8^+^ T cells were stained with CellTrace Violet (Thermo Fisher Scientific) and 2.5 × 10^5^ lymphocytes (5:1 ratio with BMDCs) were co-incubated for 48 h at 37 °C with the pre-mixed BMDCs and Raji HA-GFP tumor cells after the phagocytosis step. Cell suspensions were subsequently stained with a viability marker, anti-CD11c and anti-CD8a and T-cell proliferation assessed by flow cytometry.

### Statistical analysis

GraphPad Prism was used for all statistical analysis. The unpaired t test or One-way ANOVA test were used for statistical comparison. Data were expressed as mean ± SEM or mean ± SD, as indicated. *p*-value of < 0.05 was considered significant. *p < 0.05, **p < 0.01, ***p < 0.001, ****p < 0.0001.

## Results

### Tumor growth inhibition by NI-1701 is associated with modulation of NK cells and myeloid cell subsets in the tumor microenvironment

**Fig. 1:**
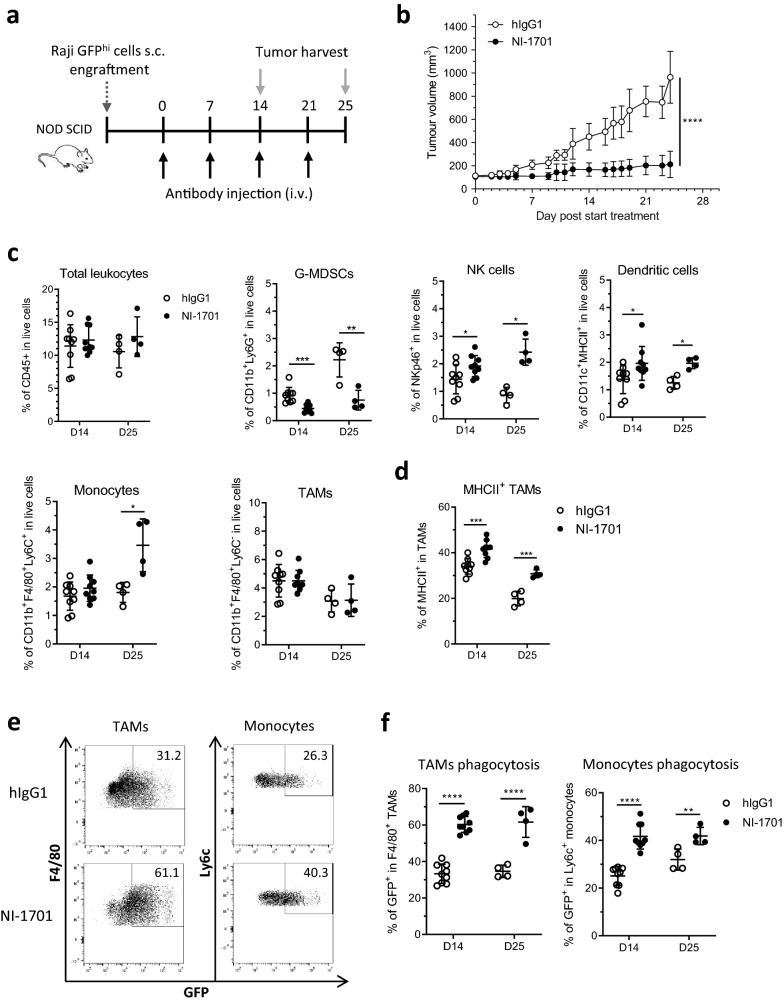
NI-1701 modulates the tumor microenvironment by promoting accumulation of immune cells associated with anti-tumorigenic functions and triggers enhanced engulfment of tumor cells by TAMs and monocytes. Immunodeficient NOD SCID mice were implanted with Raji GFP^hi^ lymphoma tumor cells and treatment was initiated when tumor volume reached 100 mm^3^. Tumors were excised 14 and 25 days after treatment initiation to evaluate impact of NI-1701 on the tumor microenvironment. **a** Design of the experiment. **b** Mean tumor volume at days 14 and 25 after treatment with human IgG1 isotype control Ab or NI-1701. Data are presented as mean ± SD, with n = 7–9 mice per group. **c** Analysis by flow cytometry of tumor-infiltrating total mouse leukocytes, myeloid cell subsets (TAMs, monocytes, G-MDSCs, dendritic cells) and NK cells. Gating strategy to identify subpopulations in the TME is displayed in Additional file 1: Fig. S1. Data are presented as mean ± SD, with n = 4–9 mice per group. **d** Analysis by flow cytometry of MHCII^+^ TAMs. **e** Representative flow cytometry plots of phagocytic TAMs or monocytes, determined as F4/80^+^GFP^+^ or Ly6C^+^GFP^+^ respectively, from Raji GFP^hi^ tumors dissected 14 days after treatment initiation. The threshold of GFP positivity and GFP internalization in TAMs and monocytes were determined based on methods and analysis depicted in supplementary experimental procedures. **f** The percentage of phagocytosis of GFP^hi^ Raji tumor cells by TAMs and monocytes, assessed at day 14 and day 25 post-treatment initiation, is shown for each individual mouse. Data are presented as mean ± SD, with n = 4–9 mice per group. Significance was determined by unpaired t test. *p < 0.05, **p < 0.01, ***p < 0.001, ****p < 0.0001

Since NI-1701 does not cross-react with mouse CD47 and CD19, we used the well-established Raji human NHL model subcutaneously implanted in immunodeficient NOD SCID mice. In this strain of mice, T- and B-cells are defective but the innate immune system, and related Fc-mediated effector cell populations, remain functional [26]. Moreover, a NOD-specific polymorphism in *Sirpa* allows a strong binding to human CD47 enabling studying this pathway in xenograft models [27]. Our previous study [24] has demonstrated that selective blockade of the CD47/SIRPα axis on Raji tumor cells with NI-1701 reduced tumor growth in vivo. To gain further insights into the mechanism of action of NI-1701, NOD SCID mice with established Raji GFP^hi^ tumors were treated with NI-1701 and tumors were excised at 2 different timepoints (D14 and D25) to characterize the immune infiltrate (Fig. 1a and Additional file 1: Fig. S1). As expected, NI-1701 inhibited tumor progression in treated mice (Fig. 1b). While no significant changes were observed in the overall percentage of CD45^+^ leukocyte infiltrate at both timepoints, as compared to hIgG1 control-treated animals, NI-1701 elicited a decrease of CD11b^+^Ly6G^+^ cells (herein referred to as granulocytic myeloid-derived suppressor cells (G-MDSCs)) with a concomitant increase of NKp46^+^ Natural Killer (NK) cells and CD11c^+^ DCs (Fig. 1c). Moreover, the proportion of CD11b^+^F4/80^+^Ly6C^+^ monocytes increased at the later timepoint whereas the percentage of CD11b^+^F4/80^+^Ly6C^−^ tumor-associated macrophages (TAMs) was not affected (Fig. 1c). As macrophages have been previously identified to be critical for antitumor activity of CD47 blocking agents, including our CD47xCD19 bsAb [9, 24], we set out to further characterize potential phenotypic changes in TAMs. Interestingly, without affecting the total macrophage percentage, NI-1701 treatment gave rise to a significant increase in MHCII^hi^ macrophages, known as M1-polarized macrophages which have a pro-inflammatory phenotype (Fig. 1d). As the tumors in NI-1701-treated mice were significantly smaller than in the control group at these timepoints (Fig. 1b), we analysed infiltrating leukocytes also at an earlier timepoint, after only 1 week of treatment (and a single dose of NI-1701), when tumor volumes were similar between the two groups (Additional file 1: Fig. S2). Consistent with our initial findings, we observed a similar trend at this earlier timepoint (Additional file 1: Fig. S2a and S2b).

Taken together, the analysis of Raji tumors revealed a NI-1701-mediated modulation of immune cell subsets in favor of an antitumor TME with enriched MHC II^+^ TAMs, NK cell and DC populations and reduced G-MDSCs.

### NI-1701 boosts the phagocytosis of tumor cells by TAMs, monocytes and dendritic cells in Raji tumors

Antibody-dependent cellular phagocytosis (ADCP) by macrophages has been shown in vitro to be a major mechanism of action of NI-1701 [24]. We thus tested whether treatment with NI-1701 would enhance ADCP of tumor cells by TAMs/monocytes in vivo but also by tumor-associated DCs, as recent reports highlighted the pivotal role of DCs at triggering antitumor T-cell response following CD47/SIRPα blockade [20, 28]. To this end, we assessed the phagocytosis events in TAMs, Ly6C^+^ monocytes (Fig. 1e and f) and CD11c^+^ DC (Additional file 1: Fig. S2c) from NI-1701-treated GFP^hi^ Raji tumors after 14 or 25 days of treatment. Remarkably, despite a high background in the hIgG1 control group, NI-1701 significantly increased tumor cell engulfment by TAMs, monocytes (Fig. 1f) and, albeit to a lesser extent, by CD11c^+^ DCs (Additional file 1: Fig. S2c) at the two time-points.

This data suggests that ADCP by tumor-associated phagocytes may play an important role in NI-1701-mediated inhibition of tumor growth in vivo.

### NI-1701 induces an immune activating transcriptome in the TME

**Fig. 2:**
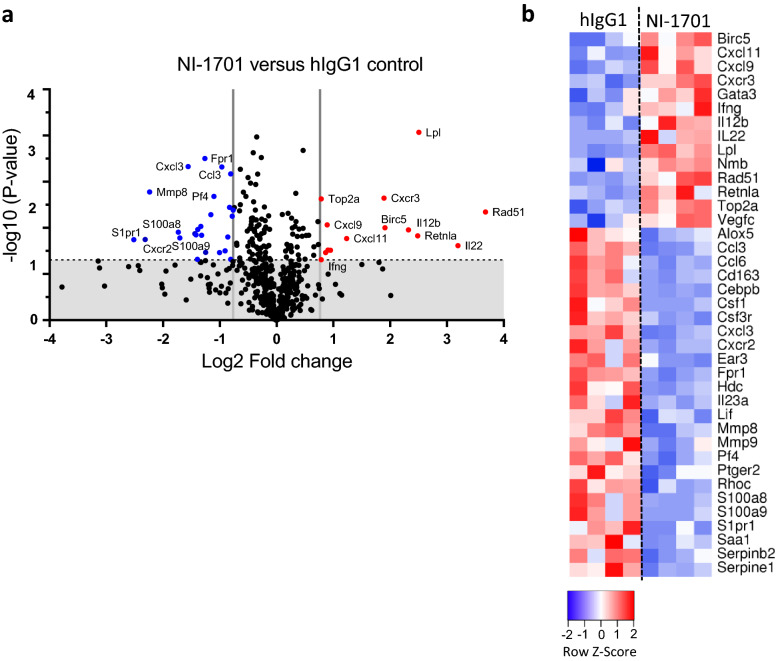
Targeted transcriptomic analysis of tumor-infiltrating mouse leukocytes reveals upregulation of proinflammatory cytokine and chemokine genes upon NI-1701 treatment. Mouse CD45^+^ tumor-infiltrating leukocytes were sorted from tumors 14 days after treatment initiation with NI-1701 or hIgG1 control and submitted to Nanostring transcriptomic analysis using the mouse Myeloid Innate Immunity panel (n = 4 mice per group) **a** Volcano plot of differentially expressed genes in tumor-infiltrating leukocytes between hIgG1 versus NI-1701. Vertical grey lines correspond to absolute log2 fold change > 0.76 (i.e., fold-change > 1.7) and horizontal dot line correspond to a *p* value of *p* = 0.05. Grey zone corresponds to statistically non-significant changes and red and blue dots to significantly upregulated and downregulated genes, respectively. **b** Heatmap depicting row *z* scores of normalized mRNA count of significantly up-regulated and down-regulated genes with fold change > 1.7

To further dissect the molecular mechanisms by which NI-1701 controls Raji tumor growth, we isolated tumor-infiltrating mouse leukocytes after 14 days of treatment and performed targeted transcriptomic analysis. Among the 734 genes in the Myeloid Innate Immunity panel, 39 genes were affected by NI-1701 treatment (Fig. 2a). 14 genes in the NI-1701-treated group were significantly up-regulated (red dots) and 25 were downregulated (blue dots) as compared to the control. Consistent with the reduction of the G-MDSCs population induced by NI-1701, several genes expressed by granulocyte such as *Cxcr2, Mmp8* and *Fpr1* were significantly down-regulated (Fig. 2b). Remarkably, an immune activation signature was observed in the NI-1701 cohort with an increased gene expression of the inflammatory cytokines *Il12b, Il-22, Ifng* as well as the IFNγ-inducible chemokine genes *Cxcl9* and *Cxcl11* (Fig. 2b), which play a key role in macrophage activation and immune cell recruitment [29].

Interestingly, we also observed a significant up-regulation of the lipoprotein lipase *Lpl* gene (Fig. 2a) which could reflect increased metabolic activity of TAMs. In fact, macrophages can produce and secrete lipoprotein lipase (LPL) to facilitate fatty acid availability as another source of fuel than glucose metabolism especially when energy requirements are increased [30, 31]. Overall, these gene transcriptional changes in tumor-infiltrating leukocytes underscore immune cell activation in the TME after NI-1701 administration.

### Macrophages and NK cells are critical effector cells for NI-1701 antitumor response in vivo

**Fig. 3:**
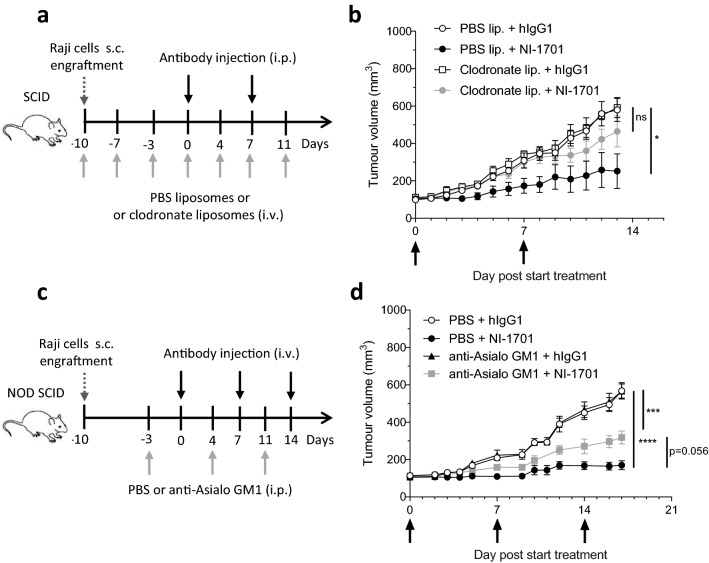
Both macrophages and NK cells are important effector cells for NI-1701 antitumor efficacy in Raji lymphoma xenograft model. **a** Design of the macrophage depletion experiment. **b** Tumor volume follow-up of CB17-SCID mice carrying Raji tumors treated with i.p. injection of NI-1701 and i.v. injection of clodronate liposomes. n = 5 mice per group. **c** Design of the NK cell depletion experiment. **d** Tumor volume follow-up of NOD SCID mice carrying Raji tumors treated with i.v. injection of NI-1701 and i.p. injection of anti-Asialo GM1 antibody. n = 8 mice per group. For **b** and **d** mean ± SEM is shown. Significance was determined at endpoint by one-way ANOVA test. *p < 0.05, ***p < 0.001, ****p < 0.0001

An increased phagocytosis of tumor cells by TAMs and an increased NK cell infiltration associated with an immune activation signature prompted us to study the importance of these two cell populations for the NI-1701-mediated control of Raji tumors. To assess their contribution to the antitumor response, we used clodronate liposomes to deplete phagocytic cells and the polyclonal Ab anti-Asialo-GM1 to deplete NK cells (Fig. 3a and c). Phagocytes depletion was performed in CB17 SCID mice as clodronate liposomes cause toxicity in NOD SCID mice [32]. A preliminary experiment demonstrated that the kinetics of Raji lymphoma tumor growth and NI-1701 antitumor efficacy in CB17 SCID mice were comparable to NOD SCID mice (data not shown). Treatment with clodronate liposomes induced a depletion of splenic macrophages, whereas depletion in the TME was inefficient (Additional file 1: Fig. S3a). However, that was enough to cause a loss of antitumor effect of NI-1701 (Fig. 3b). On the other hand, depletion of NK cells observed in both the spleen and the tumor after anti-Asialo-GM1 treatment (Additional file 1: Fig. S3b) led to a partial reduction of NI-1701 efficacy (Fig. 3d). Taken together, these results demonstrate that both macrophages and NK cells contribute to NI-1701-mediated inhibition of Raji lymphoma tumor growth.

### NI-1701 promotes DC-mediated tumor cell killing and cross-priming of CD8^+^ T cells

**Fig. 4:**
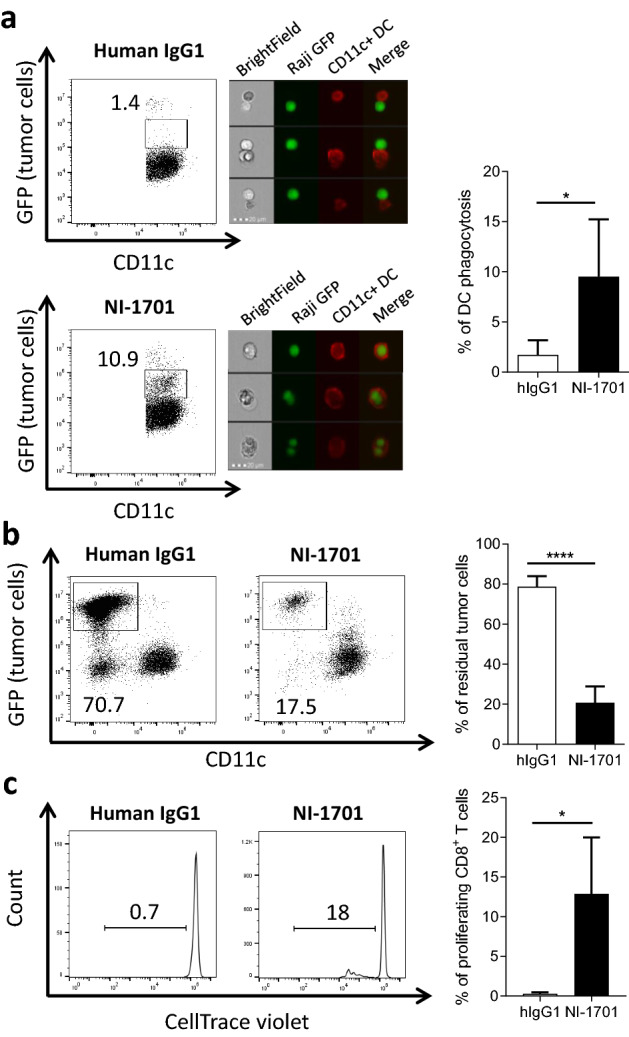
NI-1701 induces tumor cell phagocytosis by bone-marrow-derived dendritic cells and promotes cross-priming of CD8^+^ T cells. Bone marrow-derived mouse dendritic cells (CD11c^+^) from BALB/c mice were cocultured for 2 h 30 (**a**) or 24 h (**b**) with Raji GFP^hi^ tumor cells (1:1 ratio) in the presence of hIgG1 or NI-1701. Representative flow cytometry plots for hIgG1 and NI-1701 treated groups are depicted, and the percentage of phagocytosis is indicated (left panel). Phagocytic events were confirmed by imaging flow cytometry acquisition in the CD11c^+^GFP^+^ gate with tumor cells in green fluorescence and CD11c^+^ dendritic cells in red fluorescence (middle panel). The mean percentage of phagocytosis at 2 h 30 ± SD is shown (right panel), n = 5 independent experiments. **b** Percentage of residual Raji GFP^hi^ tumor cells after 24 h of coculture with CD11c^+^ DCs was determined by the analysis of remaining GFP^+^ tumor cells within the total live cell gate. n = 5 experiments. **c** Bone-marrow derived dendritic cells from BALB/c mice were cocultured overnight with hemagglutinin-expressing Raji tumor cells (Raji HA-GFP) in the presence of human IgG1 or NI-1701 (ratio 1:1). The next day, HA-specific CD8^+^ T cells from CL-4 transgenic mice (bearing HA-specific TCR on CD8^+^ T cells) labelled with CellTrace violet were added (ratio 1:5). Analysis of CD8^+^ T cell proliferation was performed 3 days later. Plots represent an illustration of proliferating CD8^+^ T cells (left panel). Mean ± SD was calculated from 4 independent experiments (right panel). Significance was determined by unpaired *t* test. *p < 0.05, ****p < 0.0001

Studies in immunocompetent animal models suggest that adaptive immune responses subsequent to CD47/SIRPα blockade are significantly involved in tumor growth control [20, 28, 33, 34]. As DCs appear to play a pivotal role in the cross-priming of antitumor T-cell response mediated by anti-CD47 mAb [28, 35], we investigated whether NI-1701-induced tumor cell phagocytosis by DCs could trigger antigen cross-presentation to T cells. DCs were derived from GM-CSF stimulated bone-marrow cells of BALB/c mice and expressed CD11c marker, MHC II, costimulatory molecules CD80 and CD86 and SIRPα were (Additional file 1: Fig. S4). In vitro phagocytosis assay with Raji GFP^hi^ cells showed higher engulfment of tumor cells by DCs after NI-1701 treatment (9.5% versus 1.7% CD11c^+^GFP^+^ events for hIgG1, Fig. 4a), which was confirmed by analysing double-positive CD11c^+^GFP^+^ cells using imaging flow cytometry. More strikingly, co-incubation of BMDCs and tumor cells in presence of NI-1701 for 24 h resulted in an almost total elimination of tumor cells (Fig. 4b). Tumor cell killing was certainly mediated by DCs, as NI-1701 alone did not induce direct cell death of tumor cells (data not shown). Finally, to investigate whether NI-1701 can influence DC antigen cross-presentation and T-cell priming, we used hemagglutinin A (HA) as a surrogate tumor-associated antigen in an in vitro cross-presentation assay. The percentage of proliferating HA-specific CD8^+^ T cells in the NI-1701 treated group was significantly higher than in the hIgG1-treated group (12.9% from NI-1701 group vs 0.4% from hIgG1 group respectively, Fig. 4c). These results demonstrated that NI-1701-treated DCs induced the priming of HA-specific T-cells after phagocytosis of HA-expressing Raji cells.

## Discussion

CD47/SIRPα innate immune checkpoint inhibitors have emerged as a new promising class of cancer immunotherapy that might synergize with treatments that invigorate antitumor T-cell response. To mitigate the safety risks, and the impact of CD47 antigen sink on pharmacokinetic profiles of CD47 targeting reagents, more selective approaches are being developed such as anti-SIRPα antibodies [36], next-generation CD47 mAbs with lower binding to red blood cells [37, 38] and CD47xTAA bsAbs [23, 39].

In this study, we aimed to elucidate the effector mechanisms behind the in vivo antitumor activity of NI-1701, a bsAb targeting CD47 and CD19 [24]. NI-1701 administration to tumor-bearing mice was shown to modify the tumor microenvironment by promoting the infiltration of immune cells, including M1-polarized inflammatory macrophages, NK cells, and CD11b^−/+^CD11c^+^ DCs, all known for their anti-tumorigenic functions [40–42]. Consistent with these findings, targeted transcriptomic NanoString analysis of tumor-infiltrating leukocytes revealed that pro-inflammatory cytokine and chemokine genes *Ifng*, *Il-12b*, *Il-22*, *Cxcl9* and *Cxcl10* were upregulated in infiltrating leukocytes from NI-1701-treated tumors. Although we have not quantified cytokines/chemokines at the protein level nor properly identified the cellular subsets responsible for the upregulation of these genes, we believe that the increased accumulation of NK cells and/or DCs may be the dominant source of IFNγ. Indeed, the aforementioned cell populations have been shown to be the major source of IFNγ in the same strain of mice used in our experiments, i.e., NOD SCID mice, following *L. monocytogenes* stimulation [43] and, thus, may contribute to the up-regulation of *Ifng* and related genes in the tumor xenograft model described herein.

On the other hand, a significant decrease of CD11b^+^Ly6G^+^ G-MDSCs was observed following NI-1701 treatment, which is in favor of an antitumorigenic microenvironment, as such immune cells can suppress innate and adaptive immune response promoting tumor growth. However, we should highlight that, as there are no phenotypic cell surface markers that allow for the separation of classical neutrophils from G-MDSCs [44], we could not rule out that the G-MDSC population in our study also contains neutrophils. In addition, the CD11b^+^Ly6C^+^ monocyte population we described could also contains M-MDSCs as the markers are also similar. Further functional assessment would be required to better differentiate these immune cell subsets.

Since tumor-cell phagocytosis by monocytes/macrophages has been extensively described as a key mechanism of action of CD47/SIRPα blocking molecules, we have explored the phagocytic activity of these cell populations in the TME. A high level of phagocytosis for both cell types (and also DCs) was observed in the control group and might be explained by physiological phagocytosis and removal of dead tumor cells that have gone through apoptosis [45]. Remarkably, NI-1701 increased the phagocytosis of tumor cells by macrophages and monocytes in the TME. We also observed that both MHC II^low^ and MHC II^hi^ TAMs subpopulations (considered as M2- and M1-like macrophages, respectively) were able to efficiently engulf tumor cells (data not shown). This is consistent with previous studies suggesting that, while M2-like or tumor-conditioned macrophages have pro-tumor properties, they maintain effective Fc-dependent phagocytic function [46–49]. Furthermore, we observed a significant upregulation of the gene encoding lipoprotein lipase *Lpl* (LPL) in isolated tumor-infiltrating leukocytes from the NI-1701 treated cohort, which we attributed to the dominant TAM subpopulation. This observation is consistent with a previously described example of *Lpl* upregulation in microglia from mice bearing glioblastoma tumors exposed to anti-CD47 mAb treatment [50]. While the exact role of LPL in TAMs is not well-established, LPL has been implicated in monocyte differentiation and macrophage accumulation [51] as well as in maintaining macrophage phagocytic activity under low glucose conditions [30]. It is likely that LPL may help to maintain TAM phagocytic activity under hypoxia conditions in the TME.

ADCP and ADCC were described as key antitumor mechanisms induced by NI-1701 in vitro [24]. To dissect the relative contribution of macrophages and NK cells to NI-1701 antitumor efficacy in vivo, cell depletion experiments were performed. Clodronate liposomes were effective at suppressing splenic macrophages, however depletion did not occur in the TME. This is consistent with previous reports showing that clodronate liposomes injected systemically efficiently deplete macrophages in spleen, bone marrow and liver, whereas macrophage suppression in subcutaneous tumors is heterogeneous and depends on the tumor model [46, 52]. However, as partial depletion of macrophages reduced the antitumor efficacy of NI-1701 to a level near that of the hIgG1 isotype control, we conclude that the monocyte/macrophage lineage plays a key role in the antitumor activity of NI-1701 in the Raji tumor model. On the other hand, effective systemic depletion of NK cells in the same model resulted in a reduction of NI-1701 activity, also supporting a contribution of NK cells to antitumor effect. As it is known that the NK cell activity in NOD SCID mice is partially impaired [26], the contribution of NK cells to NI-1701 antitumor activity may be under-estimated with this model. As NK cells were recently reported to express SIRPα upon activation, and that CD47 blockade may augment NK cell-mediated antitumor responses [10], we suspect that NI-1701 can exert an ADCC effect through both Fc-dependent mechanism and CD47/SIRPα blockade [12]. Thus collectively, we show that macrophages and NK cells may cooperate to trigger optimal tumor control following NI-1701 administration.

Originally, the antitumor activity of CD47/SIRPα axis inhibitors has been attributed to enhanced direct killing of cancer cells, principally by macrophage-mediated ADCP [12, 13]. However, CD47 blockade may also contribute to tumor elimination through promoting cytotoxic T cell responses [20, 28, 33, 53]. This idea is corroborated by the demonstration that CD47 blockade synergizes with PD-1/PDL-1 immune checkpoint inhibitors in immunocompetent mouse tumor models [20, 34, 54]. In fact, dendritic cells were described to be critical for antitumor effect of CD47 blockade in syngeneic mouse models to promote antitumor T-cell responses [28, 35]. As the xenograft model employed here does not allow the evaluation of the adaptive immune response, we studied the ability of NI-1701 to induce DC cross-priming of CD8^+^ T-cells in vitro. We showed that mouse BMDC could induce a low-level phagocytosis of tumor cells in a short time frame (2.5 h). Unexpectedly, a longer exposure to NI-1701 (24 h) resulted in almost total elimination of tumor cells over time. These results point towards an unappreciated role of DCs in direct elimination of tumor cells, probably through ADCP. To our knowledge, such a DC-mediated direct killing has not been demonstrated previously. On the other hand, tumoricidal activity of DC in vitro has already been documented and different cytotoxic mechanisms have been described including engagement of the death receptor ligand FasL, TRAIL or TNFα, or still NO pathway [55]. Whether DC killing of tumor cells mediated by NI-1701 depends primarily on the blockade of the CD47/SIRPα axis or on Fc-dependent mechanisms has not been elucidated yet and remains to be clarified. We finally demonstrated that NI-1701-induced tumor cell engulfment significantly increased the capacity of DC to promote antigen cross-presentation and antigen specific T-cell proliferation. Consistent with these data, we previously reported that mouse CD47-targeting bsAbs inhibited tumor growth in an immunocompetent lymphoma mouse model and also promoted T-cell immune response in vitro [56].

## Conclusion

Overall, this study further supports the use of the CD47xCD19 bsAb, NI-1701, as a selective CD47/SIRPα innate immune checkpoint inhibitor to mobilize innate immunity for the treatment of B-cell malignancies. NI-1701 is currently being evaluated in a Phase I clinical trial for the treatment of refractory or relapsed B-cell lymphoma (NCT04806035).

## Supplementary Information

**Additional file 1. **Supplementary figures.

**Additional file 2. **Supplementary methods.

## Data Availability

All the data relevant to the study results are included in the main figures of the article or available in the additional files. The datasets used and analyzed during the study are available from the corresponding author on reasonable request.

## Abbreviations

Ab: Antibody
ACK: Ammonium–Chloride–Potassium
ADCC: Antibody-dependent cellular cytotoxicity
ADCP: Antibody-dependent cellular phagocytosis
bsAb: Bispecific antibody
BMDC: Bone-marrow-derived dendritic cell
CD: Cluster of differentiation
CRISPR: Clustered regularly interspaced short palindromic repeats
DC: Dendritic cells
Fc: Fragment crystallizable
FcγR: Fc gamma Receptor
GFP: Green fluorescent protein
GM-CSF: Granulocyte-macrophage colony-stimulating factor
G-MDSCs: Granulocytic myeloid-derived suppressor cells
HA: Hemagglutinin
IFNγ: Interferon gamma
IgG: Immunoglobulin G
IRES: Internal ribosome entry site
mAb: Monoclonal antibody
MHC: Major histocompatibility complex
NHL: Non-Hodgkin lymphoma
NK: Natural Killer
NO: Nitric oxide
s.c.: Subcutaneous
SD: Standard deviation
SEM: Standard error mean
SIRPα: Signal regulatory protein alpha
TAM: Tumor-associated macrophage
TME: Tumor microenvironment
