# Supplementary material for: CD47xCD19 bispecific antibody triggers recruitment and activation of innate immune effector cells in a B-cell lymphoma xenograft model
Source: Exp Hematol Oncol. 2022 May 10;11:26. doi: 10.1186/s40164-022-00279-w (PMC9088114; doi:10.1186/s40164-022-00279-w)
Supplement: Supplementary file 1 — Additional file 1. Supplementary figures. [file 40164_2022_279_MOESM1_ESM.pptx]

## Slide 1
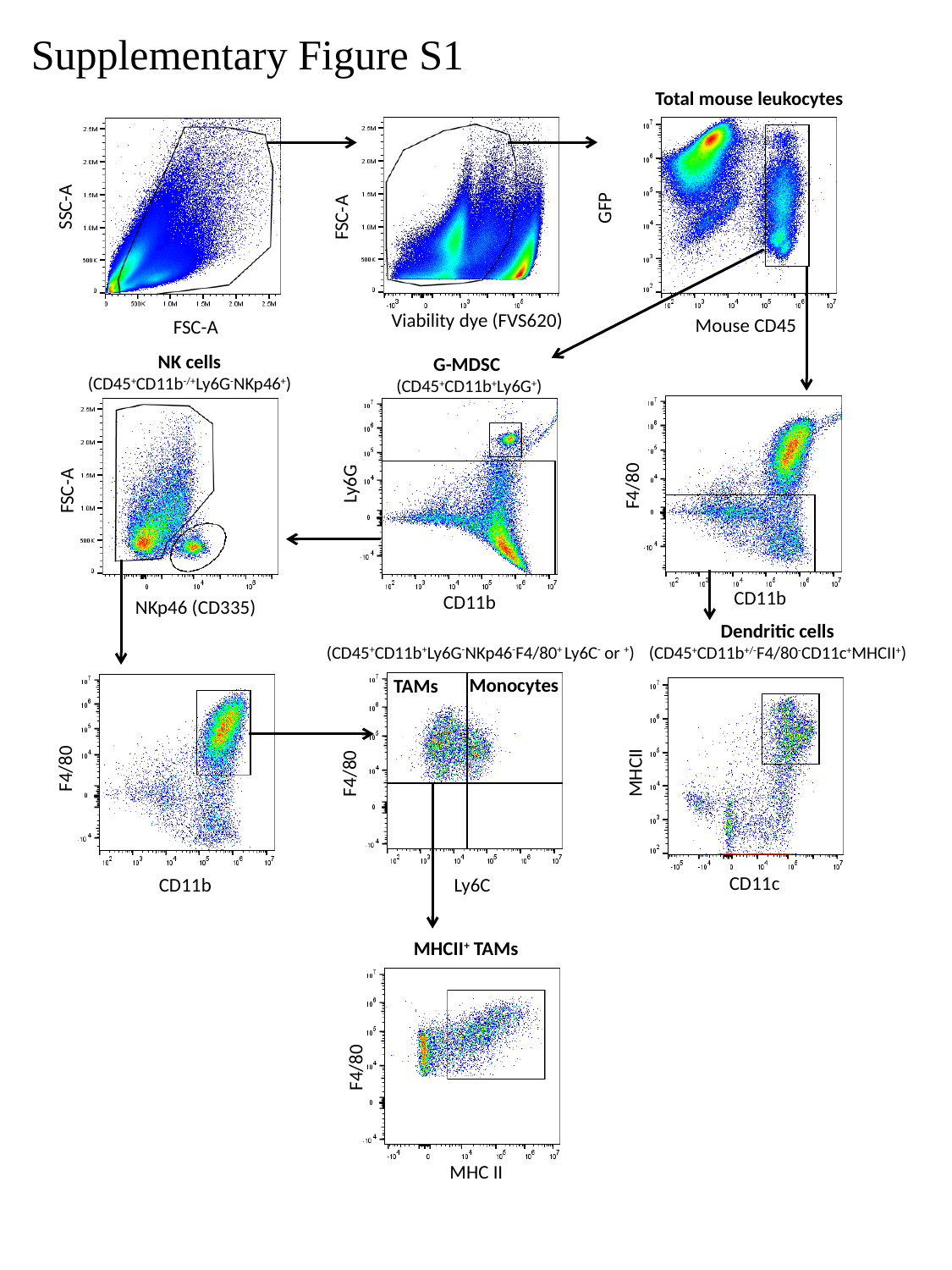

Supplementary Figure S1
Total mouse leukocytes
SSC-A
GFP
FSC-A
Viability dye (FVS620)
Mouse CD45
FSC-A
G-MDSC
(CD45+CD11b+Ly6G+)
NK cells
(CD45+CD11b-/+Ly6G-NKp46+)
Ly6G
F4/80
FSC-A
CD11b
CD11b
NKp46 (CD335)
Dendritic cells
(CD45+CD11b+/-F4/80-CD11c+MHCII+)
(CD45+CD11b+Ly6G-NKp46-F4/80+ Ly6C- or +)
Monocytes
TAMs
F4/80
MHCII
F4/80
CD11c
Ly6C
CD11b
MHCII+ TAMs
F4/80
MHC II

## Slide 2
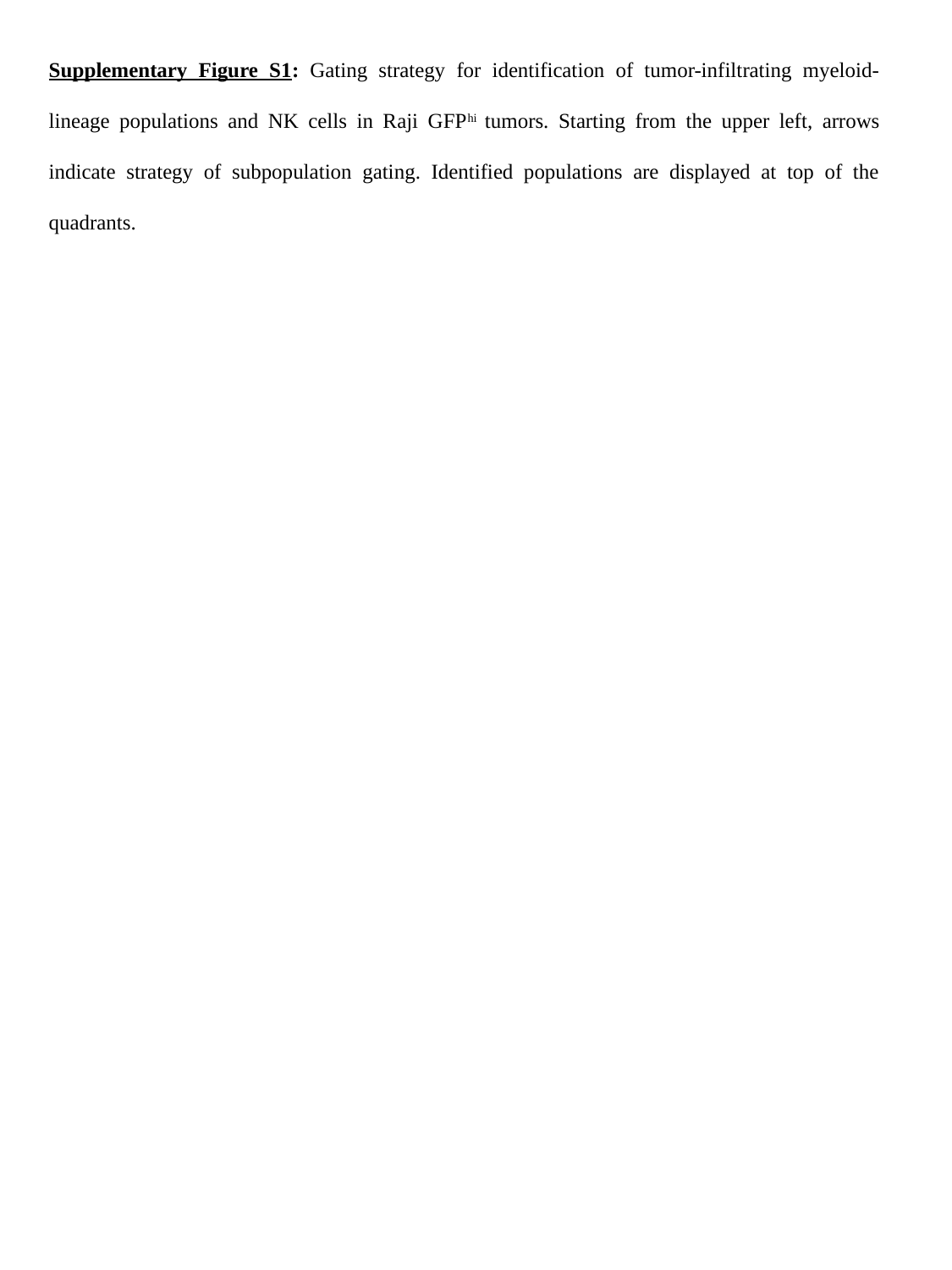

Supplementary Figure S1: Gating strategy for identification of tumor-infiltrating myeloid-lineage populations and NK cells in Raji GFPhi tumors. Starting from the upper left, arrows indicate strategy of subpopulation gating. Identified populations are displayed at top of the quadrants.

## Slide 3
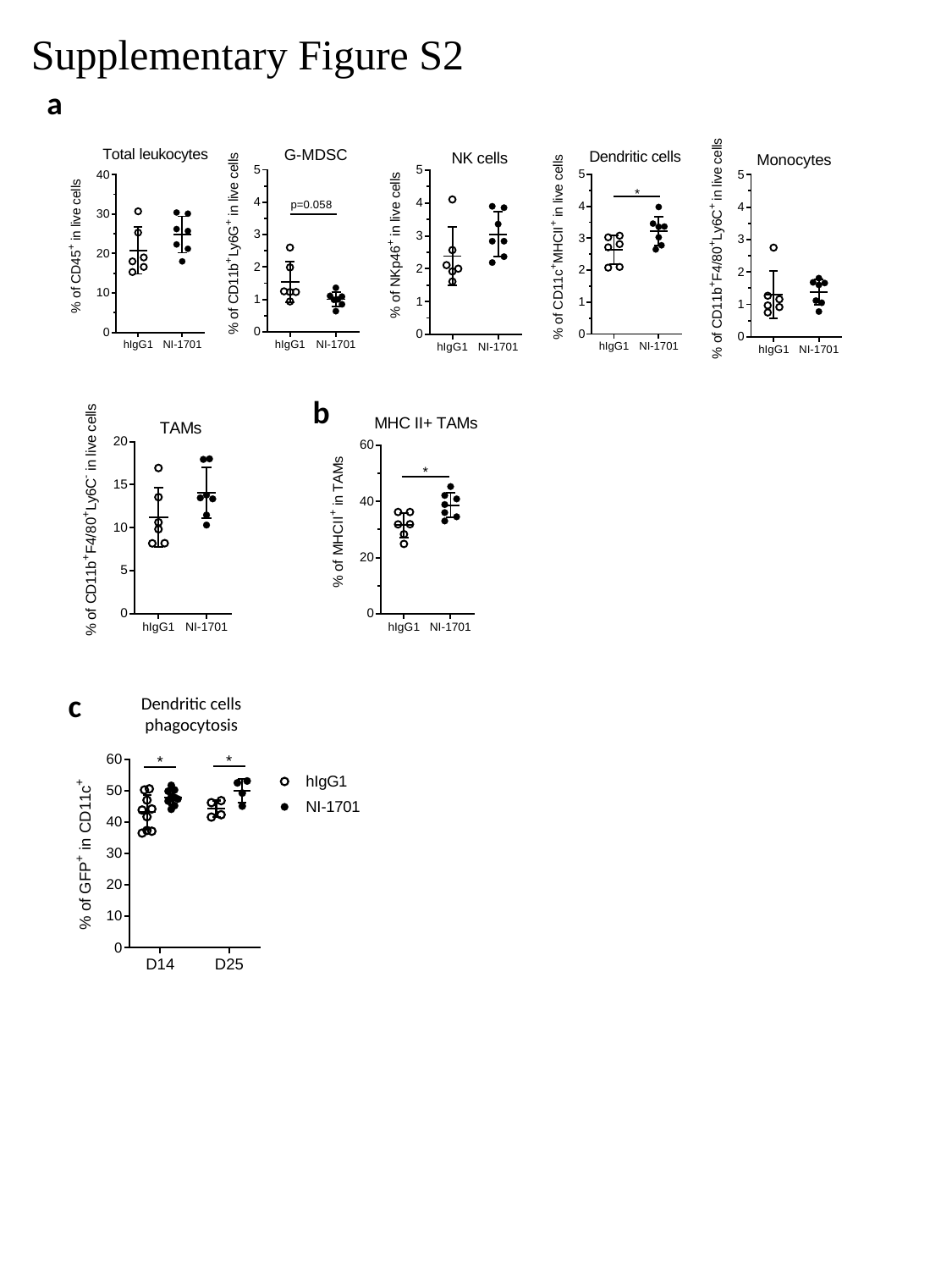

Supplementary Figure S2
a
b
c
Dendritic cells
phagocytosis

## Slide 4
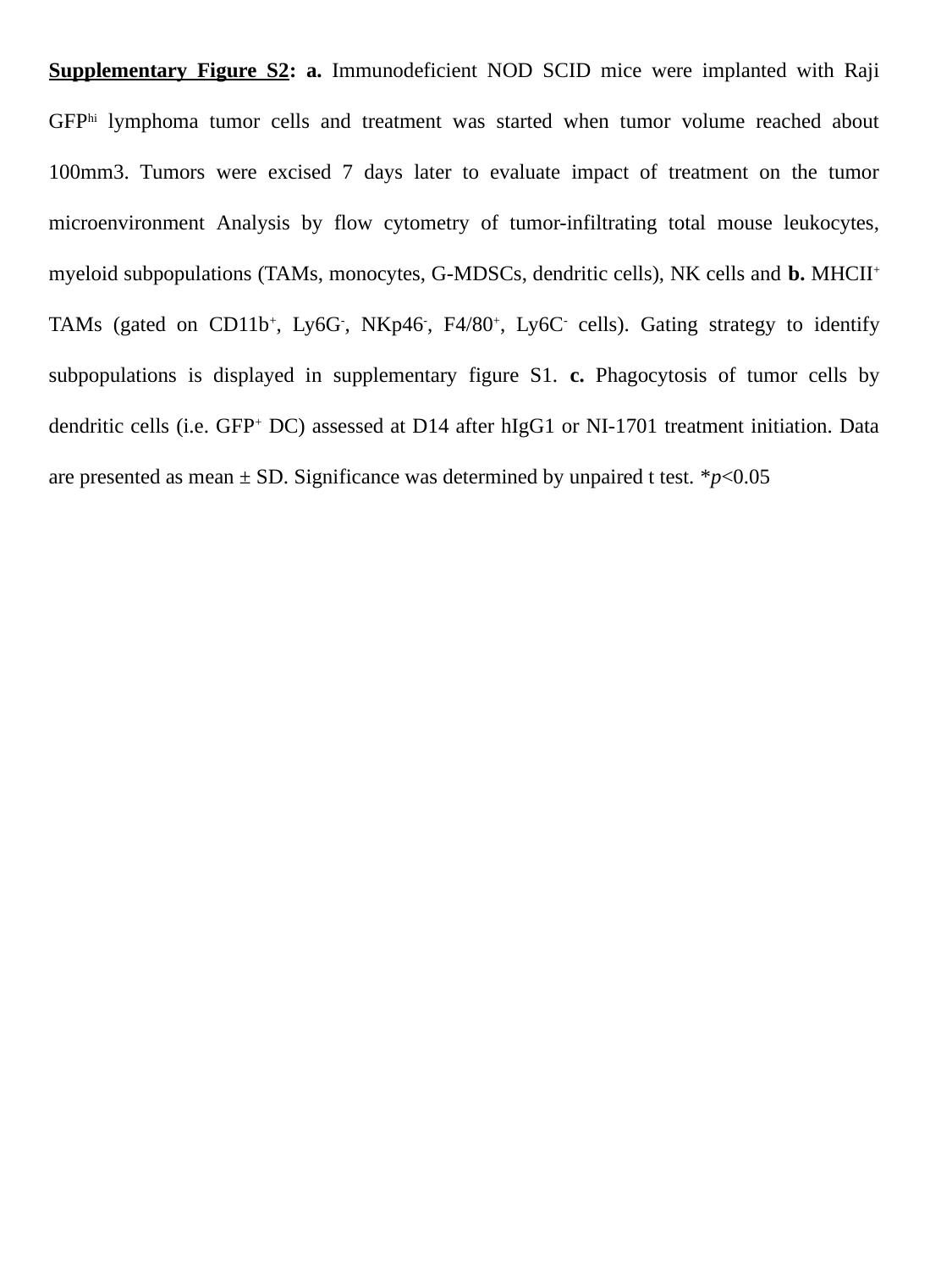

Supplementary Figure S2: a. Immunodeficient NOD SCID mice were implanted with Raji GFPhi lymphoma tumor cells and treatment was started when tumor volume reached about 100mm3. Tumors were excised 7 days later to evaluate impact of treatment on the tumor microenvironment Analysis by flow cytometry of tumor-infiltrating total mouse leukocytes, myeloid subpopulations (TAMs, monocytes, G-MDSCs, dendritic cells), NK cells and b. MHCII+ TAMs (gated on CD11b+, Ly6G-, NKp46-, F4/80+, Ly6C- cells). Gating strategy to identify subpopulations is displayed in supplementary figure S1. c. Phagocytosis of tumor cells by dendritic cells (i.e. GFP+ DC) assessed at D14 after hIgG1 or NI-1701 treatment initiation. Data are presented as mean ± SD. Significance was determined by unpaired t test. *p<0.05

## Slide 5
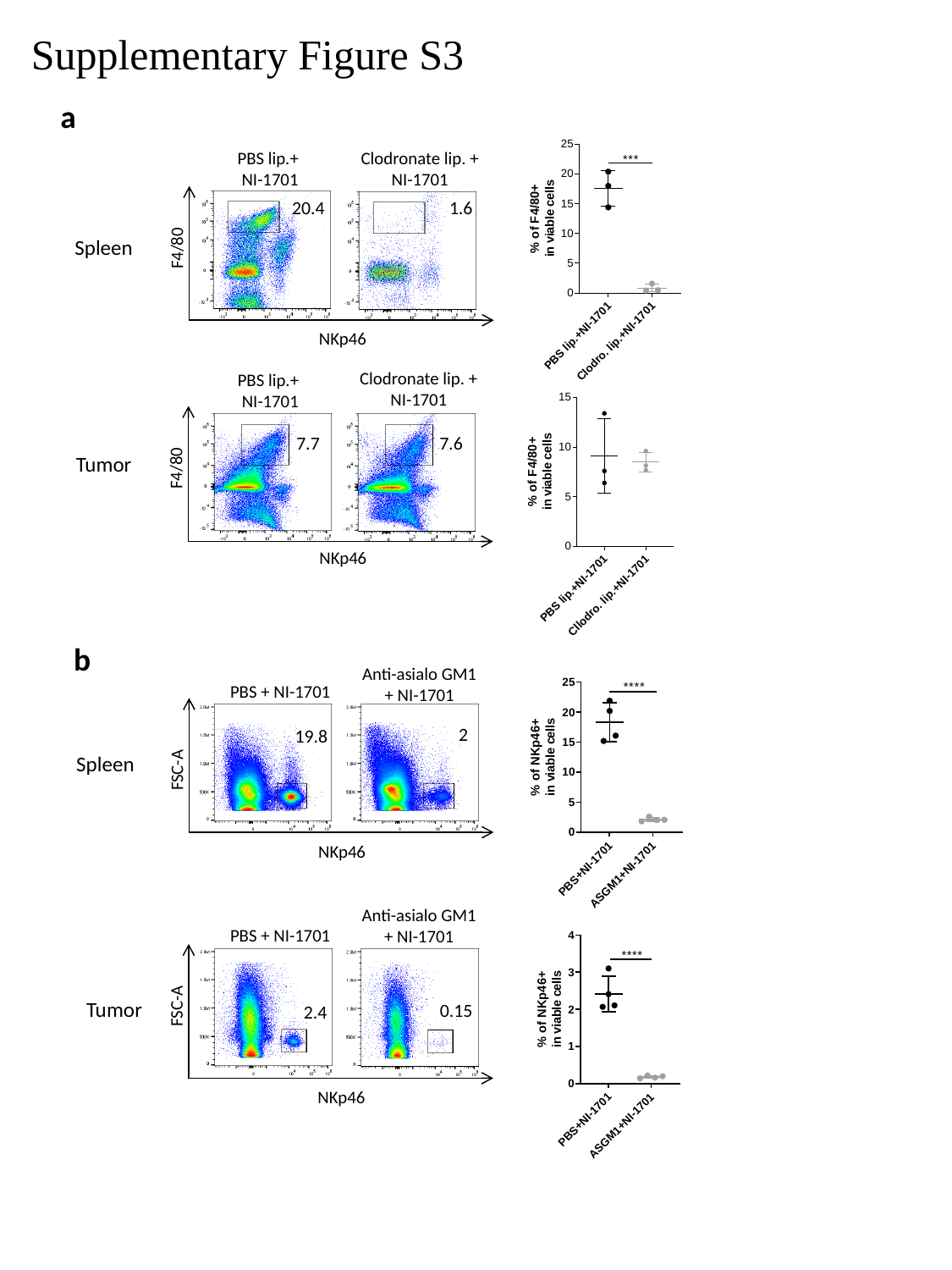

Supplementary Figure S3
a
PBS lip.+ NI-1701
Clodronate lip. + NI-1701
1.6
20.4
Spleen
F4/80
NKp46
Clodronate lip. + NI-1701
PBS lip.+
NI-1701
7.7
7.6
Tumor
F4/80
NKp46
b
Anti-asialo GM1
+ NI-1701
PBS + NI-1701
2
19.8
Spleen
FSC-A
NKp46
Anti-asialo GM1
+ NI-1701
PBS + NI-1701
FSC-A
Tumor
0.15
2.4
NKp46

## Slide 6
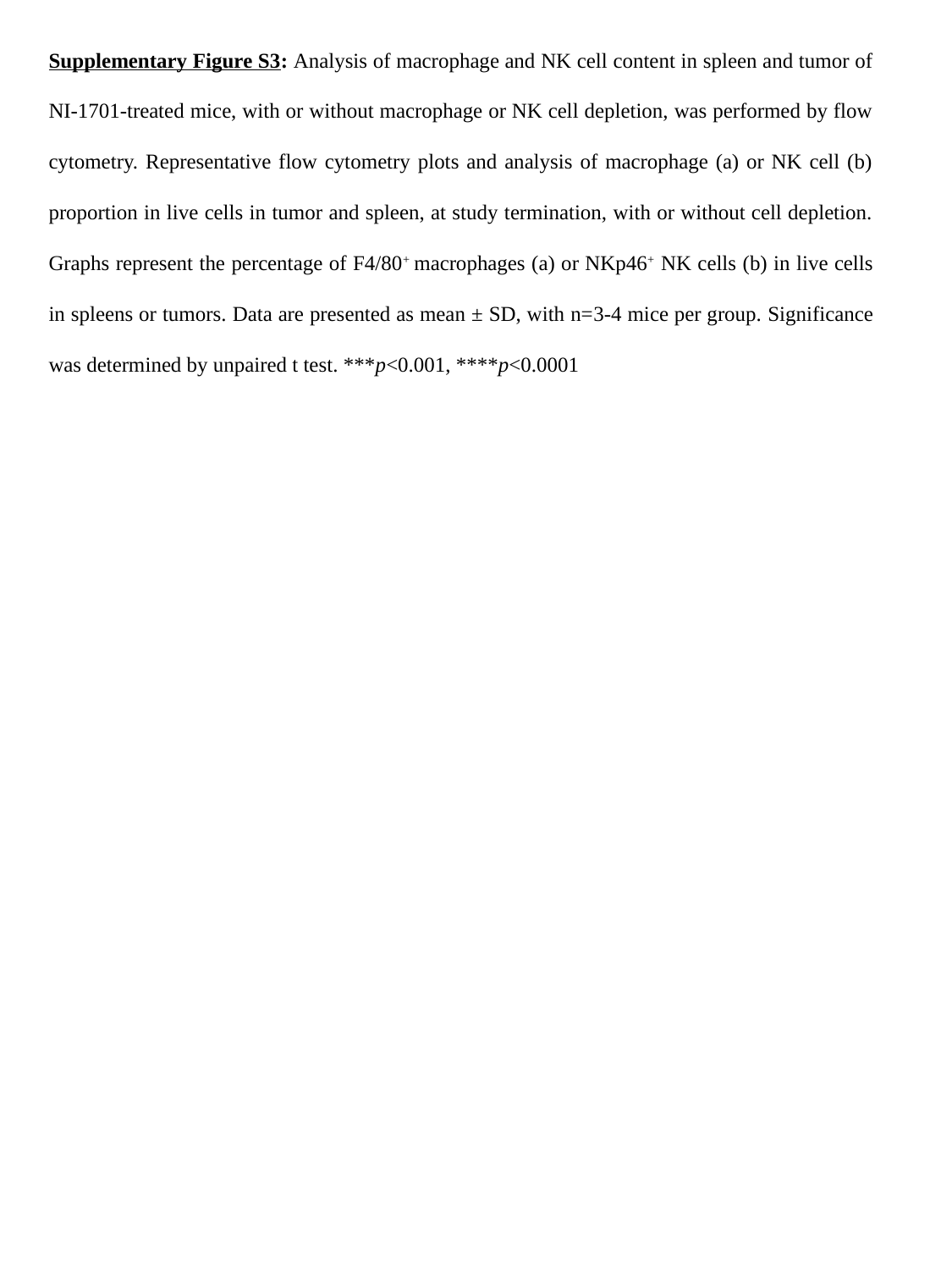

Supplementary Figure S3: Analysis of macrophage and NK cell content in spleen and tumor of NI-1701-treated mice, with or without macrophage or NK cell depletion, was performed by flow cytometry. Representative flow cytometry plots and analysis of macrophage (a) or NK cell (b) proportion in live cells in tumor and spleen, at study termination, with or without cell depletion. Graphs represent the percentage of F4/80+ macrophages (a) or NKp46+ NK cells (b) in live cells in spleens or tumors. Data are presented as mean ± SD, with n=3-4 mice per group. Significance was determined by unpaired t test. ***p<0.001, ****p<0.0001

## Slide 7
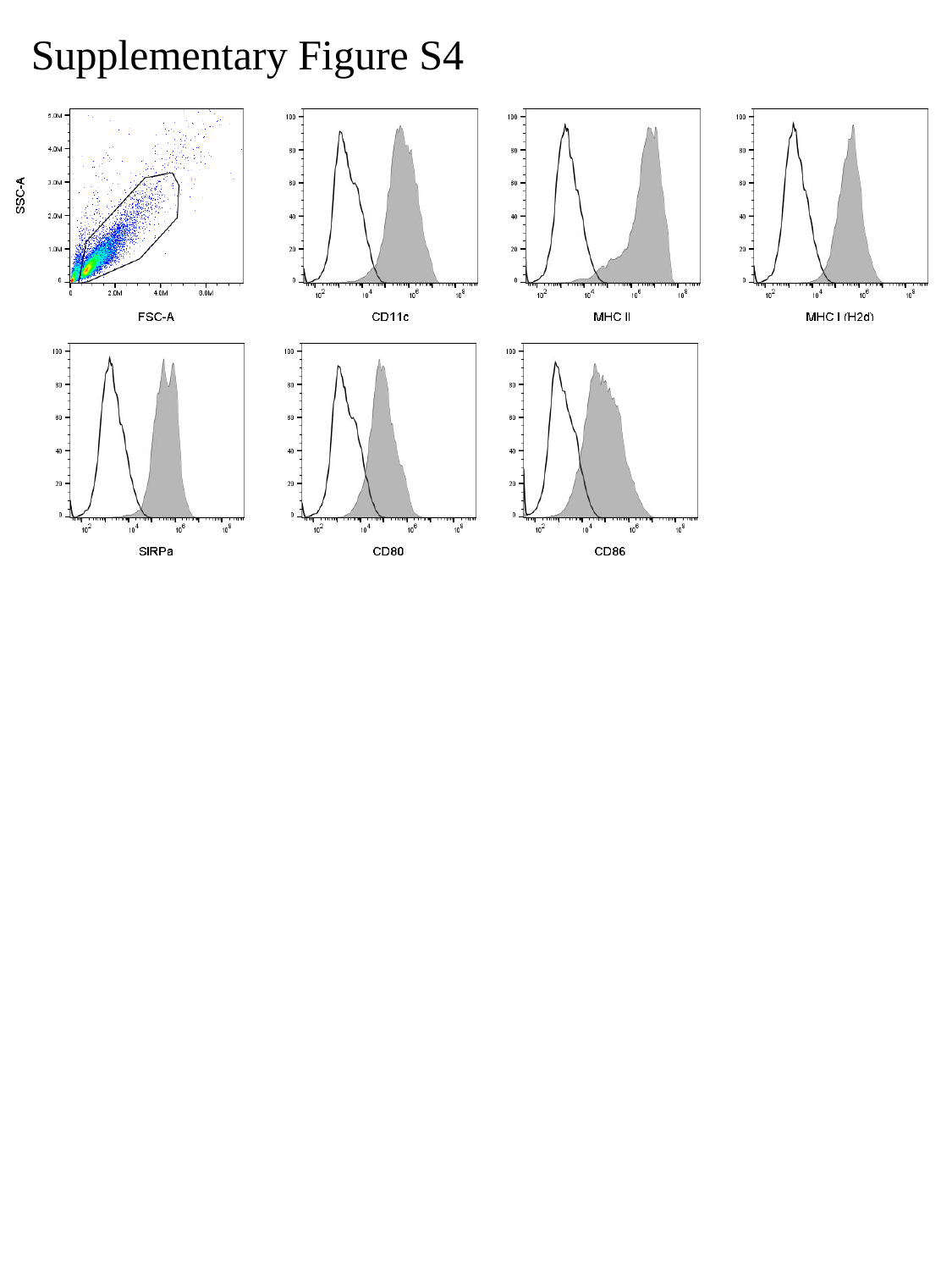

Supplementary Figure S4

## Slide 8
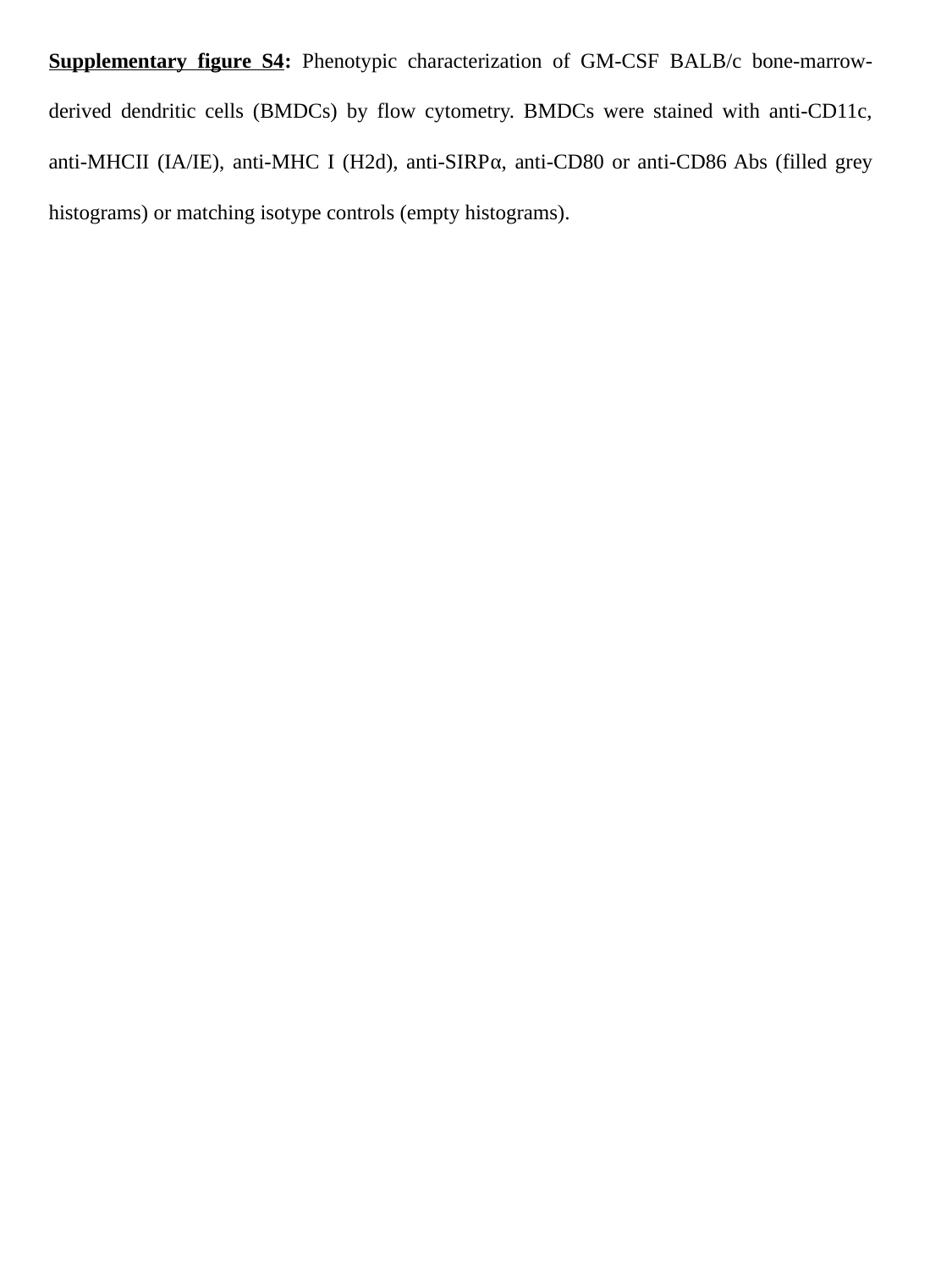

Supplementary figure S4: Phenotypic characterization of GM-CSF BALB/c bone-marrow-derived dendritic cells (BMDCs) by flow cytometry. BMDCs were stained with anti-CD11c, anti-MHCII (IA/IE), anti-MHC I (H2d), anti-SIRPα, anti-CD80 or anti-CD86 Abs (filled grey histograms) or matching isotype controls (empty histograms).
