# Supplementary material for: CD47xCD19 bispecific antibody triggers recruitment and activation of innate immune effector cells in a B-cell lymphoma xenograft model
Source: Exp Hematol Oncol. 2022 May 10;11:26. doi: 10.1186/s40164-022-00279-w (PMC9088114; doi:10.1186/s40164-022-00279-w)

**Supplementary Methods**

**Imaging flow cytometry analysis (FlowSight, Luminex Corp.): gating and masking strategy to validate *in vivo* tumor cell phagocytosis by macrophages and dendritic cells**

*Gating strategy:*

Gradient RMS_M01_Ch01 was used to find focused cells. Focused cells were then gated based on Area_M01 versus Aspect Ratio_M01 (size vs. circularity) to gate single cells. This population of single-focused cells was used for downstream analysis. First, live mouse CD45^+^ Ly6G^-^ NK^-^ cells were divided into macrophages (F4/80^+^) and DC (F4/80^-^ CD11c^+^) populations. GFP positive populations were then gated based on GFP negative tumor cells before applying masks and calculate percentage of GFP internalization.

*Example of masking strategy:*

The tables describe the set-up of the macrophage cytoplasm mask and GFP green zone mask allowing the analysis of the internalized tumor cell (Raji GFP^+^) in macrophages.

| **Cytoplasm mask** | **Mask definition** | **Description** | **Image** |
| --- | --- | --- | --- |
| Submask 1 | M01 | Default mask on BrightField image |  |
|  | Erode(M01, 3) | The Erode function removes 3 pixels from all edges of the starting mask to remove membrane |  |
| Submask 2 | M07 | Default mask on Ch07 (mouse CD45) image (target cells are negative) |  |
|  | Intensity(M07, Ch07, 150-4095) | The Intensity function sets a range of fluorescence intensity to remove background signal |  |
|  | Fill(Intensity (M07, Ch07, 150-4095)) | The Fill function fills any hole in the starting mask |  |
|  | Erode(Fill(Intensity(M07, Ch07, 150-4095)), 1) | The Erode function removes 1 pixel from all edges of the starting mask |  |
| Combined submasks | Erode(M01, 3) And Erode(Fill(Intensity(M07, Ch07, 150-4095)), 1) | Only the cytoplasm of mCD45+ cells is masked |  |

| **Total green zone mask** | **Mask definition** | **Description** | **Image** |
| --- | --- | --- | --- |
|  | M02 | Default mask on Ch02 image |  |
|  | Intensity(M02, Ch02, 100-4095) | The Intensity function sets a range of fluorescence intensity to remove background signal |  |

| **Green zone in cytoplasm mask** | **Mask definition** | **Description** | **Image** |
| --- | --- | --- | --- |
|  | Cytoplasm | Cytoplasm mask defined above |  |
|  | Intensity(Cytoplasm, Ch02, 100-4095) | The Intensity function sets a range of fluorescence intensity to remove background signal |  |

Final masking parameters for analysis:

-Cytoplasm: Erode (M01, 3) And Erode(Fill(Intensity(M07, Ch07, 150-4095)), 1)

-Total green zone: Intensity (M02, Ch02, 100-4095)

-Green zone in the cytoplasm of macrophage: Intensity(Cytoplasm, Ch02, 100-4095)

*Imaging flow cytometry picture galleries and visual inspection of masking strategy:*

Values displayed in black on Bright Field (BF) image corresponds to the object number, and values displayed in yellow on the GFP/mCD45 picture corresponds to the percentage of GFP signal falling into the mouse CD45^+^ (mCD45) macrophage or DC cytoplasm (Formula: 100 * Area_Green zone in cytoplasm / Area_Total green zone). A threshold of 80% of GFP signal into mCD45^+^ cells was fixed using picture galleries of GFP^+^ macrophages and DCs to discriminate real phagocytosis (≥ 80%) from associated events (˂ 80%).

Analysis of the whole GFP^+^ events demonstrated that the large majority of GFP^+^ DC or GFP^+^ macrophages correspond to real phagocytosis events (i.e. ≥ 80% of the GFP signal of the tumor cells being inside the macrophages or DC).


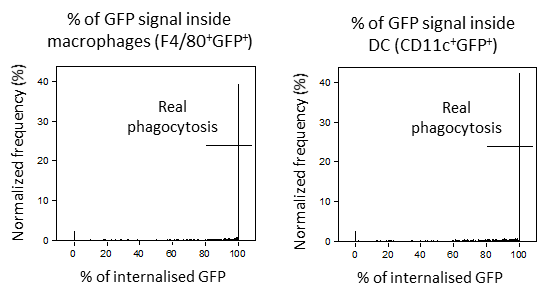

Supplement: Supplementary file 2 — Additional file 2. Supplementary methods. [file 40164_2022_279_MOESM2_ESM.docx]
